# Shorter Exposures to Harder X-Rays Trigger Early Apoptotic Events in *Xenopus laevis* Embryos

**DOI:** 10.1371/journal.pone.0008970

**Published:** 2010-01-29

**Authors:** JiaJia Dong, Sean P. Mury, Karen E. Drahos, Marko Moscovitch, Royce K. P. Zia, Carla V. Finkielstein

**Affiliations:** 1 Integrated Cellular Responses Laboratory, Department of Biological Sciences, Virginia Polytechnic Institute and State University, Blacksburg, Virginia, United States of America; 2 Department of Physics, Virginia Polytechnic Institute and State University, Blacksburg, Virginia, United States of America; 3 Department of Radiation Medicine, Georgetown University Medical Center, Washington D. C., United States of America; National Cancer Institute, United States of America

## Abstract

**Background:**

A long-standing conventional view of radiation-induced apoptosis is that increased exposure results in augmented apoptosis in a biological system, with a threshold below which radiation doses do not cause any significant increase in cell death. The consequences of this belief impact the extent to which malignant diseases and non-malignant conditions are therapeutically treated and how radiation is used in combination with other therapies. Our research challenges the current dogma of dose-dependent induction of apoptosis and establishes a new parallel paradigm to the photoelectric effect in biological systems.

**Methodology/Principal Findings:**

We explored how the energy of individual X-ray photons and exposure time, both factors that determine the total dose, influence the occurrence of cell death in early *Xenopus* embryo. Three different experimental scenarios were analyzed and morphological and biochemical hallmarks of apoptosis were evaluated. Initially, we examined cell death events in embryos exposed to increasing incident energies when the exposure time was preset. Then, we evaluated the embryo's response when the exposure time was augmented while the energy value remained constant. Lastly, we studied the incidence of apoptosis in embryos exposed to an equal total dose of radiation that resulted from increasing the incoming energy while lowering the exposure time.

**Conclusions/Significance:**

Overall, our data establish that the energy of the incident photon is a major contributor to the outcome of the biological system. In particular, for embryos exposed under identical conditions and delivered the *same* absorbed dose of radiation, the response is significantly increased when shorter bursts of more energetic photons are used. These results suggest that biological organisms display properties similar to the photoelectric effect in physical systems and provide new insights into how radiation-mediated apoptosis should be understood and utilized for therapeutic purposes.

## Introduction

Programmed cell death, or apoptosis, is a central cellular process in normal cell turnover, tissue homeostasis, stress response signaling, aging, and in maturation of the immune system [1], [2], [3]. Perturbation of signaling cascades regulating apoptosis results in an imbalanced apoptotic rate that leads to profound effects on the whole organism and can initiate a wide variety of human diseases [4], [5], [6], [7]. Apoptotic signals, both intracellular and extracellular, converge to activate a group of apoptosis-specific proteases termed caspases, a family of cysteine proteases with specificity for aspartic acid residues in their substrates [8]. Interestingly, irrespective of the genotoxic stimuli, death results in the same apoptotic morphology that includes pyknosis, extensive plasma membrane blebbing, DNA cleavage to nucleosome-sized fragments, and caspase-mediated cleavage of cellular proteins [1], [3]. This observation suggests a cascade mechanism for transmission of signals, the extent of which is not fully known although it converges in a predictable, relatively small number of reactions.

Exposure of cells to physiological and environmental stress conditions, such as radiation, temperature changes, nutrient limitation, hypoxia, oxidative stress, and exposure to carcinogens, tumor promoters, chemical mutagens, or oncogenic viruses results in an adaptive response that impacts cell cycle progression, death, survival, and differentiation [9]. Specifically, genotoxic stress induced by DNA damaging agents, such as ionizing (X- or gamma-) radiation and radiomimetic drugs, leads to several types of DNA lesions including modifications such as 8-oxoguanine formation, single- and double-strand breaks, DNA base-pairing mismatches, and abnormal cross-links in DNA or between DNA and cellular proteins (for review see [10]). Such alterations induce genetic instability resulting in a number of different phenotypes including senescence, necrosis, apoptosis, chromosome damage, and mitotic catastrophe [11]. Radiation-induced apoptosis has been studied extensively in organs and established cell lines, further determining that cells react to injuries causing DNA damage in different ways, depending on both the type and dose of irradiation [12]. A dose-dependent increase in apoptosis was observed in mice thymocytes treated with doses above 0.2–0.5 Gy [13], and in actively proliferating osteosarcoma spheroids in response to doses of ionizing radiation of 5 and 30 Gy [14]. In addition, dose- and time-dependent induction of apoptosis was observed in the endothelium of the brain and spinal cord [15]. Further studies show that small intestine cells exhibit a remarkable sensitivity to radiation [16], [17]. Dose as low as 0.01 Gy resulted in a significant increase of apoptotic cells per crypt compared with the number of spontaneous events occurring in the same proliferative unit of the small intestine (for review see [18]). Interestingly, the spatial distribution of apoptotic cells in the crypt remained the same even when variable sources of radiation such as gamma-ray or neutrons and dose rates were tested [19].

Adverse consequences of radiation exposures depend on the amount of DNA damaged as determined by the absorbed dose, expressed in terms of energy absorbed per unit weight and measured as joules/kg (named Gy) as well as by its form as determined by the linear energy transfer (LET, keV/µm), with low LET radiation for X- and gamma-rays and beta particles [20], [21], [22]. Here, the dose is directly related to the total energy delivered by the beam, *E*, which is the product of *three* factors: *ε*, the energy of individual photons (in the case of X- or gamma-rays), *j*, the rate at which photons impact the sample, and *T*, the exposure time. This raises the question of whether a biological system is then intrinsically more sensitive to changes in any one of the specific dose parameters. Whereas this hypothesis remains to be proven in a biological setting, it is a well-characterized phenomenon in physics known as the photoelectric effect. In the photoelectric effect, the intensity of a light beam (X- or gamma-ray) is not the key quantity controlling the existence of an electric current. In other words, exposing a surface to a certain “dose” of radiation (*E*) during the course of an experiment does not determine whether electrons will emerge or not from the irradiated surface. To explain this effect, Einstein hypothesized that a beam consists of a stream of photons, each of which carries a unit of energy (ε), which is inversely proportional to the incident wavelength (λ); thus ε = *hc*/λ where *h* is Planck's constant and *c* is the speed of light. In this context, the “dose” is simply equal to *E = *ε × *j* × *T*. However, the most unexpected discovery from the photoelectric effect is that *only* ε, rather than *simply E*, determines electron emission. In particular, if *j* remains constant throughout the experiment while ε and *T* vary accordingly to keep the same “dose” *E*, the result is dramatic: electrons are emitted only when a surface is exposed for a short time (small *T* values) to energetic (large ε values) photons, whereas long exposures of low energy photons will cause no effect.

Here, we hypothesize that damage (evidenced by apoptosis) to biological samples also follows the properties of the photoelectric effect. For example, cell death should be more pronounced in embryos exposed to X-rays with higher energy (ε) for short intervals than those experiencing lower energy for longer periods of time, even when total exposure doses are essentially the same. To test this hypothesis, we exposed embryos to X-rays with a range of energies for various periods of time, guided roughly by the assumptions that *i*) the current (*j*) is constant and *ii*) the average energy of the X-rays (ε) is proportional to the voltage setting (in units of *kV*). Under these assumptions, the product of the voltage (*kV*) and exposure time (*T*) is an estimate of the total dose. To ensure that these assumptions are valid, we used thermoluminescence dosimetry (TLD) to measure directly total doses (Gy) *absorbed* by the embryos. Our findings strongly suggest that, for embryos exposed to essentially the *same dose*, the photon energies of the X-ray play a significant role in inducing apoptosis.

## Methods

### Ethics Statement

The *Xenopus laevis* (XENOUPS EXPRESS, Inc.) embryos study received written ethical approval from the Institutional Animal Care and Use Committee at Virginia Tech. All proposals involving the use of living vertebrates at Virginia Tech comply with: U.S. Government Principles for the Utilization and Care of Vertebrate Animals Used in Testing, Research, and Training; The Animal Welfare Act, as amended; The Public Health Service (PHS) Policy on Humane Care and Use of Laboratory Animals and Virginia Tech Policies Governing the Use of Animals in Research and Teaching.

### Preparation of Embryos

Eggs were fertilized *in vitro* as described previously [23] and embryos were staged according to Nieuwkoop and Faber [24]. For time-course experiments, embryos were irradiated at stage 6 (morula), collected at the indicated times, frozen on dry ice, and stored at −80°C. Irradiation was performed by exposing stage 6 embryos to various energies from a TFI Mini Shot X-ray machine for the indicated times. Embryos were visualized with an Olympus SZX-ILLB2-100 stereo microscope and photographed with an Olympus Camedia C-5060 digital camera.

### Assay of Apoptosis in a Cell-Free System

This assay was performed according to conditions described previously [25], [26] with the following modifications. Embryos were irradiated at stage 6 and collected at different times after irradiation. In substrate cleavage assays, ^35^S-labeled *Xenopus* cyclin A2 translated *in vitro* (TNT-coupled reticulocyte lysate system, Promega) was added at a 1∶10 dilution into an extract volume equivalent to one embryo. Samples were incubated at 30°C, and aliquots of 3 µl were withdrawn at various times and diluted with 6x SDS-PAGE sample buffer. The cleavage products were resolved by SDS-PAGE and visualized by autoradiography.

### In Vitro Caspase Assay

DEVDase caspase activity (Caspase 3/7 Glo, Promega) assays were performed according to manufacturer's instructions in a 96-well white plate format and analyzed using a Beckman Coulter LD400 plate reader. Embryos were homogenized in EB buffer [20 mM Hepes pH 7.5, 80 mM β-glycerophosphate, 20 mM EGTA, 15 mM MgCl_2_, 1 mM DTT, 50 mM NaF, 1 mM sodium vanadate and 1 tablet/50 ml of protease inhibitor cocktail (GEHealthsciences)] and processed for caspase analysis as described [26]. Briefly, aliquots of embryo extracts (10 µg) were incubated with 100 µl of Caspase-Glo 3/7 substrate for 1 h at room temperature. Luminescence was measured at 492 nm.

### Whole-Mount TUNEL (TdT-Mediated dUTP-X Nick End Labeling) Assay

Double-stranded breaks in DNA were detected as described [27]. Albino embryos treated with different doses of ionizing radiation were collected when development appeared abnormal and controls were gastrulating (st.8+4 h). Briefly, embryos were fixed in MEMFA (100 mM Mops pH 7.4, 2 mM EGTA, 1 mM Mg_2_SO_4_, 3.7% formaldehyde) for 1–2 h at room temperature, dehydrated, and stored in ethanol at −20°C. Embryos were rehydrated through an ethanol series and sequentially washed with PBS, PBST (0.2% Tween 20 in PBS), and terminal deoxynucleotidyl transferase (TdT) buffer (Invitrogen). Embryos were then incubated with 150 U/ml of TdT (Invitrogen) and 0.5 µ*M* digoxygenin–dUTP (GE Healthsciences) overnight at room temperature. The reaction was terminated by incubation of the embryos in PBS containing 1 mM EDTA for 1 h at 65°C, followed by washes in PBS at room temperature. Detection and chromogenic reaction was carried out as described [28]. Embryos were blocked in PBT containing 20% goat serum, followed by incubation with alkaline phosphatase-conjugated anti-digoxygenin Fab fragment (GE Healthsciences). After extensively washing the embryos in PBS for 24 h, specimens were stained using nitro blue tetrazolium and 5-bromo-4-chloro-3-indolyl phosphate substrates. The color reaction was visible within 30 min, and embryos were photographed after re-fixation in MEMFA for 3 h followed by dehydration in ethanol.

### Radiation Dosimetry

TLD cards were used as radiation dosimeters in this study. Following irradiation, the TLD cards were read in a Harshaw Model 8800 card reader (Thermo Fisher Scientific, Oakwood Village, Ohio, USA) [29]. Briefly, the reader incorporates a linear time–temperature controlled hot gas heating technique. The linearity of the heating profile is maintained and is directly controllable through closed loop feedback to a pre-specified maximum temperature, time, and heating rate. Although the reader can use either nitrogen or air for heating the TL elements, we decided to use highly purified dry nitrogen in order to minimize the noise in the system. The gas enters the system through four flow controls and flow meters to ensure proper flow and pressure. The gas is heated as it flows through electrical resistance heating tubes and is applied to the TL elements through nozzles located close (3 mm) to the TL element encapsulation material. Each TLD card consists of four LiFMg,Ti hot-pressed TLD chips, each of them mounted between two PTFE® films and mounted on an aluminum substrate. Since all the dose measurements were done at high dose levels where LiF∶Mg,Ti is known to exhibit supralinear dose-response, the dosimeters were calibrated at dose levels similar to the expected values during the actual experiment. This approach removed the need to apply supralinearity corrections and eliminated the uncertainty associated with supralinearity in the application of LiF∶Mg,Ti to high dose dosimetry. Furthermore, the calibration was done using low energy x-rays in order to minimize any bias associated with the energy dependence of this material.

The experimental set up for the dosimetry measurements is shown in Fig. S1.C. Briefly, embryos were placed on top of a TLD card submerged in a shallow amount of 0.1x MMR buffer (0.5 mM Hepes pH 7.8, 10 mM NaCl, 0.2 mM KCl, 0.1 mM MgSO_4_, 0.2 mM CaCl_2_) and covered by a thin layer of plastic wrap (Fig. S2.A, middle panel labeled “bottom”). Top cards were placed in the same position as the embryos to provide a good estimate for the total dose absorbed by the sample (Fig. S2.A, right panel labelet “top”). Three measurements for each experimental condition were obtained and correspond to the T1–T3 chips shown in Fig. S2.A, left panel. Of note is that the *relative* doses are similar to those for the “top” cards. In particular, even for these “bottom” cards, the readings for the 30–60 *kV* cases are quite comparable, laying within 10% of the average value (Fig. S2.B). Dose values were obtained based on the calibration of the instrument considering that 30 *kV* for 10 min is equivalent to a dose of 37 Gy.

## Results

**Figure 1:**
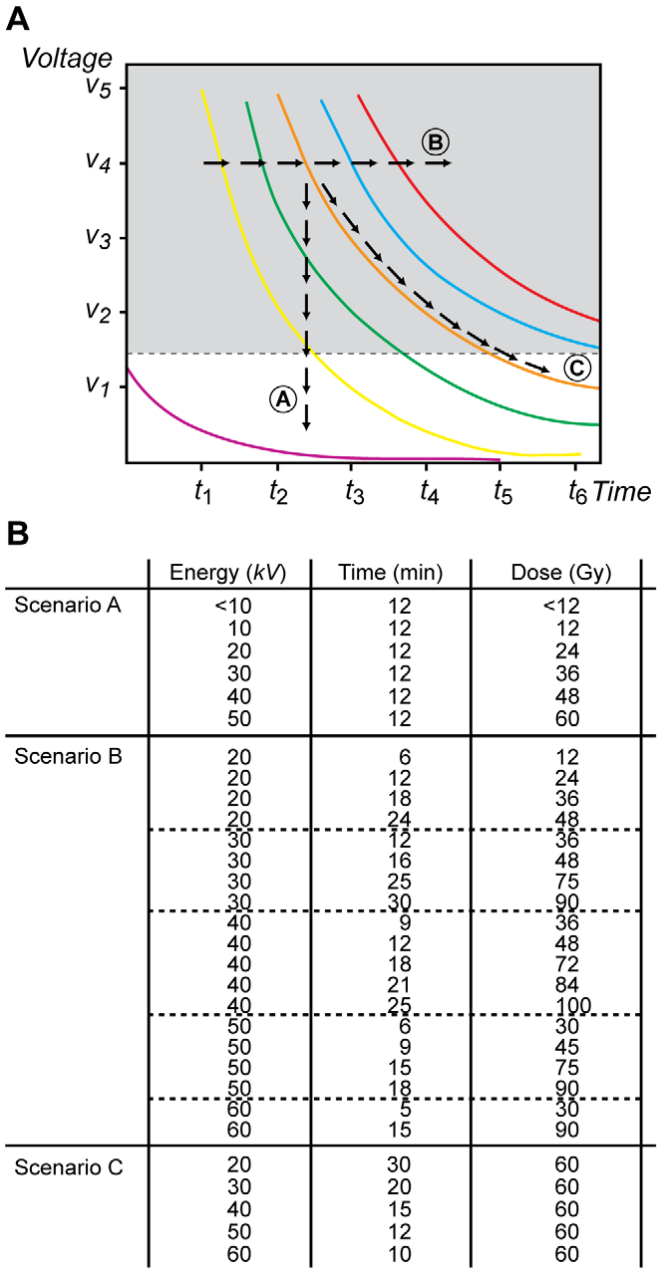
The energy-dependent hypothesis of apoptosis induction. **A.** Schematic representation of different paths in the space of control parameters (*kV*, *T*) used in our experiments. Here, we assumed that the energy of each photon (ε) generated by the X-ray source increases along with the voltage setting (*kV*) whereas its current (*j*) remains fixed. Thus, the total exposure (*E*) at which an embryo is subjected at any given time is proportional to *kV* x *T*. Three different scenarios are denoted and were tested: (Scenario A) *E* increases a as result of augmenting the energy of the photon ε and, therefore, *kV* while maintaining the exposure time (*T*) constant; (B) *E* increases by augmenting *T* while keeping a constant value for *kV*; and (C) *E* remains constant throughout all states analyzed as a result of increasing the exposure time (*T*) while diminishing *kV* accordingly. Each colored line represents a different total exposure, also named “dose”, *E*. The dotted line indicates a hypothetical threshold level below which there is not an observable effect of radiation in a biological system. **B.** Summary of the experimental conditions to be analyzed in order to evaluate the scenarios discussed above.

Most studies of radiation-induced apoptosis have centered on the mechanisms that trigger the damage response system in the cell, and less on the physical properties of the genotoxic agent. Accordingly, research on radiation-induced apoptosis has traditionally focused on the biological effect of the total dose delivered to a given system while overlooking the individual contributions of the various components of the dosage. Radiation, in the form of X-ray emission, has a characteristic energy determined by the frequency of the light. Indeed, the same total dose (*E*) delivered to a system can be achieved by a range of incident photon energies (ε), the photon flux (*j*), and the time (*T*) of exposure. Thus, in the photoelectric effect, an increase in *E alone* does not guarantee an increase in the energy of each emitted electron. Instead, electron energies increase only with the photon *frequency* (ε) above a certain threshold. On this basis, we hypothesize that cell death results from the delivery of radiation with frequencies above some threshold, and not *merely* from the total dose absorbed. To analyze the contribution of these factors to radiation-induced apoptosis *in vivo*, we have examined how various radiation scenarios impacted cell death processes in the early development of *Xenopus laevis* (Fig. 1.A). We chose *Xenopus* embryos because *i*) ionizing radiation-induced apoptosis has been extensively characterized in this specie, *ii*) pre-mid-blastula transition (MBT), but not post-MBT, irradiated embryos undergo apoptosis, *iii*) morphological and biochemical hallmarks of apoptosis including the presence of membrane-bound apoptotic bodies (blebbing), internucleosomal DNA fragmentation, pyknotic and condensed nuclei, loss of intracellular attachments, caspase activation and cleavage of specific substrates have been established in this animal model, and *iv*) embryos are tractable to a number of manipulations [26], [30], [31], [32].

Our experiments focused on three specific scenarios (Fig. 1.A). First, we evaluated the occurrence of apoptosis in embryos subjected to increasing incident energies during a fixed exposure time (Fig. 1.B). Second, we monitored the response of the biological system when the exposure time was increased while a constant energy value was maintained (Fig. 1.B). Lastly, we analyzed whether the embryos' response to the same total dose of radiation varied as a result of increasing the energy while reducing the exposure time (Fig. 1.B). A summary of the experimental conditions in each scenario is presented in Fig. 1.B.

### Scenario A: Response of Embryos to Radiation Under Constant Exposure Time

Our previous results showed that embryos irradiated at any stage before the MBT fail to repair the damage and irreversibly undergo cell death [26], [30]. Interestingly, irradiated embryos only exhibit apoptotic hallmarks after the MBT, suggesting that the MBT may be the first checkpoint monitoring developmental progression in early embryos.

**Figure 2:**
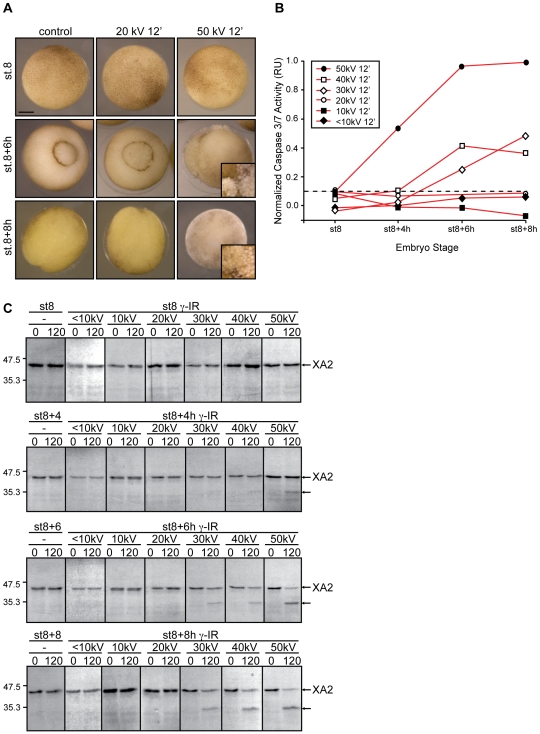
Apoptosis results from exposure to increased energies. **A.** Morphology of *Xenopus* embryos that are non-irradiated (control) or irradiated (γ-IR) with either 20 *kV* or 50 *kV* for 12 min and collected at MBT (st.8) and 6 and 8 h after. Inset shows a higher magnification image of a typical apoptotic morphology from a similar embryo. Scale bar, 250 µm. **B.** Embryos were irradiated or not (control) before the MBT (st.6) with the indicated amount of energy (<10 *kV*, 10 *kV*, 20 *kV*, 30 *kV*, 40 *kV*, 50 *kV*) for 12 min, collected at st.8 (MBT) and 4, 6 and 8 h after the MBT, and frozen. At the indicated times, samples equivalent to ten embryos were tested for the activity of caspases 3/7 using a specific colorimetric substrate as described in the “Materials and Methods” section. Normalized caspase activity refers to the activity of irradiated samples from which the basal control activity has been subtracted at each time and is expressed in relative units (RU). Points indicate the average of ten embryos at each time stage. The figure shows data from a single experiment that was repeated three times with similar results. The dotted line denotes a threshold of caspase activity from which experimental values falling below correlate with embryo samples lacking an apoptotic response. **C.** Extracts equivalent to ten embryos collected at the indicated times were incubated with radiolabeled cyclin A2 as described in the “Materials and Methods” section. At the indicated times (0 and 120 min), aliquots were removed and analyzed for cyclin A2 cleavage by SDS-PAGE and autoradiography. Control samples correspond to non-irradiated embryos. Arrows on the right denote radiolabeled *Xenopus* cyclin A2 (XA2) and its cleaved form. Molecular mass markers (in kDa) are indicated on the left.

Initial experiments were devoted to determining the relevance of the incoming energy to cell fate in early embryos. Stage 6 *Xenopus* embryos (st.6) were irradiated with voltages ranging from less than 10 *kV* to up to 50 *kV* for a fixed exposure time (12 min). Irradiated and control embryos were collected after irradiation at stage 8 (MBT; ∼6 h post-fertilization) and 4, 6, and 8 hs after the MBT and assayed for caspase 3/7 activity. Results show that embryos irradiated with energies up to 20 *kV*, with total equivalent doses up to 16 Gys, exhibited normal gastrulation and neural plate formation (∼stage (st.) 8+8 h, Fig. 2.A) whereas those treated with higher energies (*i.e.*, 50 *kV*, 12 min) displayed severe and distinct phenotypic abnormalities that correlated with the appearance of apoptotic cells [30], [32]. We then explored the functional relationship between incident energy at a constant time and apoptosis by analyzing the activity of endogenous caspases in embryos irradiated (or not irradiated, control) before the MBT and collected at different times.

First, we determined a threshold level of caspase activity that existed at a given time within the cell, which, if surpassed, would inevitably lead to uncontrolled cell death (Fig. 2.B, dotted line). Activity was assayed in extracts from control and irradiated samples using a specific colorimetric substrate as described [26]. Our data show undetectable levels of caspase activity throughout the time course analyzed when embryos were exposed to energies up to 20 *kV* for times up to 60 min (Figs. 2.B and S1). Remarkably, embryos challenged with energies of 50 *kV* (>60 Gy) exhibited apoptotic hallmarks as early as 4 h post-MBT whereas those exposed to energies equal to or greater than 30 *kV* show apoptotic features but with much slower kinetics. Thus, it seems unlikely that exposure time directly contributes to triggering apoptosis since embryos that were irradiated with different energies for a given time period and, therefore, diverse total dosages, exhibited variable biological responses.

Caspase-mediated cleavage of *Xenopus* cyclin A2 (XA2) at the ^87^DEPD^90^ (^87^Asp-Glu-Pro-Asp^90^) site removes both the destruction box and the Cdk inhibitor (CKI)-binding motif, leading to the formation of a complex, which is both insensitive to degradation and inhibition by CKIs and shows broader substrate specificity [26]. This prominent feature of the cyclin A2-cleaved complex results in expanded substrate recognition, an event that mediates DNA fragmentation during apoptosis in *Xenopus* embryos [26]. To examine the integrity of cyclin A2 in treated embryos, samples were exposed to various energies for 12 min and collected at the indicated times (Fig. 2.C). Cyclin A2 fragments were first detected in extracts from embryos exposed to 60 Gy (50 *kV*, 12 min) 4 h after the MBT and later in samples irradiated with 36 (30 *kV*, 12 min) and 48 Gy (40 *kV*, 12 min). In agreement with our caspase activity assay (Fig. 2.B), cyclin A2 cleavage was not detected in samples treated with doses below 24 Gy and with energies up to 20 *kV* (Fig. 2.C). Overall, the data presented here establish the existence of an energy threshold, equivalent to roughly 10–20 *kV* in our setup system, that needs to be surpassed before embryos irreversibly undergo apoptosis. To our knowledge, these data provide the first indication of the existence of a parallel of the photoelectric effect in biological systems.

### Scenario B: Response of Embryos to Radiation Under Constant Energy Values

**Figure 3:**
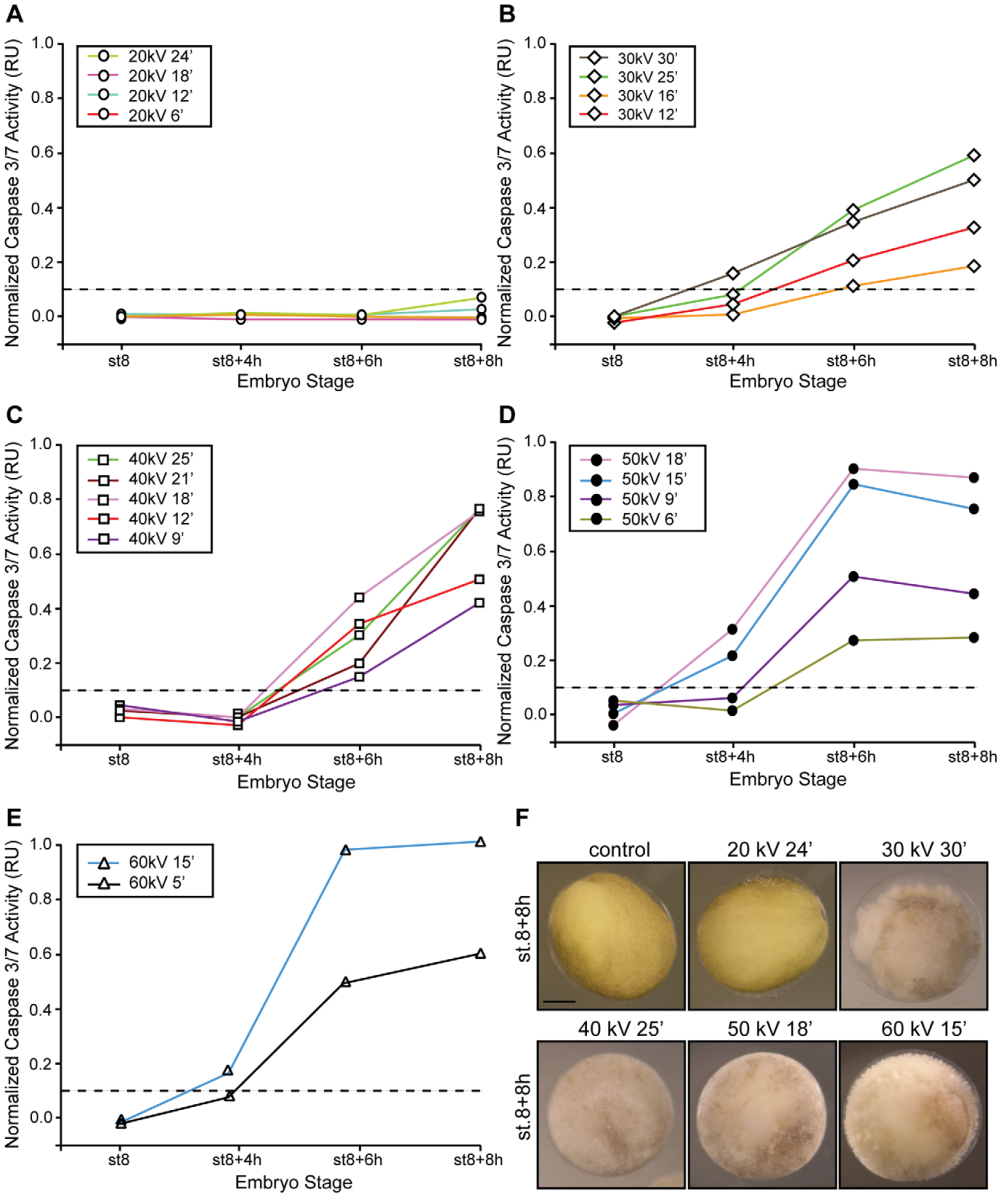
A minimum energy value is required to trigger apoptosis. Embryos were irradiated (γ-IR) or not (control) before the MBT (st.6) with either 20 *kV* (**A**), 30 *kV* (**B**), 40 *kV* (**C**), 50 *kV* (**D**) or 60 *kV* (**E**) of energy for the indicated times, collected at st.8 (MBT) and 4, 6 and 8 h after the MBT, and frozen. Samples equivalent to ten embryos were tested for caspases 3/7 activity using a specific colorimetric substrate as described in the “Materials and Methods” section and normalized as described in the legend of Fig. 2. Points indicate the average of ten embryos at each time stage. Results similar to those presented here were observed in three independent experiments. **F.** Morphology of *Xenopus* embryos not irradiated (control) or irradiated with 20, 30, 40, 50 *kV* for 24, 30, 25, 18, 15 min, respectively, and collected 8 h after the MBT. Scale bar, 250 µm.

Next, we examined whether the length of the exposure time influenced the response of the embryo to a given radiation energy. Radiation from 20, 30, 40, 50, and 60 *kV* were applied to embryos for various times and samples collected at the MBT (st.8), early gastrulation (∼st.8+4 h), and neurula stage (∼st.8+8 h) and evaluated for gross morphology and caspases 3/7 activity. Our results show that embryos irradiated with an incident energy of 20 *kV* for 6, 12, 18, and 24 min remained phenotypically unaltered and the caspase activity levels undetectable throughout the time course analyzed (Fig. 3.A). However, a number of specifics are notable. First, embryos irradiated with energies between 30–50 *kV* showed levels of caspase activity above the threshold as early as 4 h after the MBT (Figs. 3.B–E). Second, embryos exposed to energies of 40–60 *kV* for longer times (≥15 min) exhibited the greatest values of caspase activity compared with 30 *kV*-irradiated samples (Figs. 3.C–E). Third, embryos exposed for times equal to or greater than 15 min with energies of 50 and 60 *kV* showed signs of apoptosis as early as 4 hs after the MBT (Figs. 3.D–E). Accordingly, caspase activity was detected at 4 h, peaked after gastrulation and remained constant throughout the time course analyzed (Figs. 3.D–E). Interestingly, samples irradiated with low energies (30–40 *kV*) showed a linear increase in caspase activity until control embryos reached the neurula stage (Figs. 3.B–C). Lastly, embryos exposed to energies equal to or greater than 40 *kV* exhibited an exposure-time-dependent increase of caspase activity. Thus, the longer the sample was exposed to a specific energy the higher the detected caspase activity at a given time (Figs. 3.C–E). Yet, for energies of this magnitude, the system eventually proceeds to cell death even though caspase activity appeared to depend on the length of the exposure time (Figs. 3.A–F).

**Figure 4:**
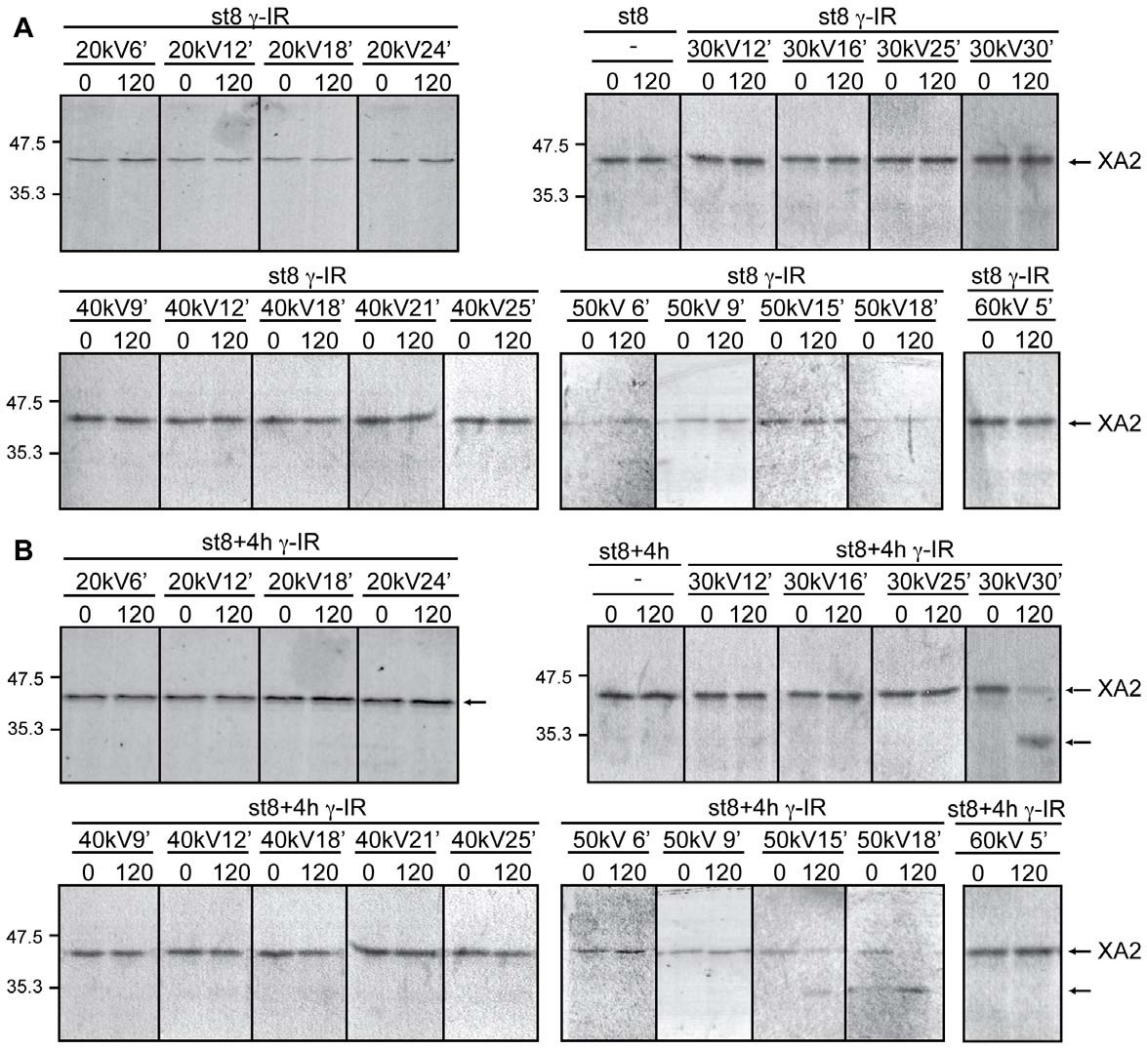
High-dose radiation raises caspase activity and favors cyclin A2 cleavage. Extracts equivalent to ten embryos from non-irradiated (control) or irradiated (γ-IR) samples collected at MBT (st.8, **A**) and 4 h after the MBT (**B**) were incubated with radiolabeled cyclin A2 as described in the “Materials and Methods” section. Aliquots were removed at the indicated times and analyzed for cyclin A2 cleavage by SDS-PAGE and autoradiography. Control samples correspond to non-irradiated embryos. Arrows on the right denote radiolabeled *Xenopus* cyclin A2 (XA2) and its cleaved form.

Cleavage of radiolabeled cyclin A2 added to extracts also supported caspases 3/7 activation in response to various radiation conditions (Fig. 4). Extracts from embryos irradiated with 20–60 *kV* for various times were incubated with labeled cyclin A2 and samples were analyzed as described in the Materials and Methods section. We decided to monitor cyclin A2 cleavage in extracts of irradiated embryos obtained from two developmental stages MBT (st.8) and early gastrulation (∼st.8+4 h). We chose these stages because at the MBT, development becomes more complex as transcription initiates, the cell cycle lengthens, and cells differentiate and organize during gastrulation. It is precisely at gastrulation when the embryo takes complete control over cell division. In addition, results presented in Fig. 3 clearly establish that embryos irradiated with doses equal to or greater than 30 *kV* exhibit caspase activity values above the threshold by 6 h after the MBT; thus, a subtle response to radiation is conspicuous only at early times.

In agreement with the data summarized in Fig. 3, extracts from embryos irradiated with various energies and for different times collected at the MBT (st.8) exhibited levels of caspase activity below a control threshold and, therefore, were unable to trigger cyclin A2 cleavage (Fig. 4, top two panels). However, when those embryos reached early gastrulation, their biological response varied depending on the energy level and time at which they were exposed. Concurrent with the results presented in Fig. 3, extracts from early gastrula embryos were able to promote cyclin A2 cleavage at energies of 30 *kV* only after 30 min exposure and with 50 *kV* at exposure times longer than 15 min. As expected, cyclin A2 levels remained steady when the labeled protein was incubated with extracts from embryos irradiated with 20 *kV* of energy for variable times (Fig. 4, lower two panels).

Therefore, our results establish that the exposure time does not determine the response of the embryo to radiation since samples treated with energies equal to or greater than 30 *kV* for roughly the same time ultimately undergo cell death (st.8+8), albeit with different kinetics. These results enforce the concept of a biological behavior that parallels the photoelectric effect in which, one of the parameters (*i.e.*, photon energies, ε) play a more fundamental role in determining the response of the system to radiation.

### Scenario C: Response of Embryos to Radiation Under Constant Total Dose Values

**Figure 5:**
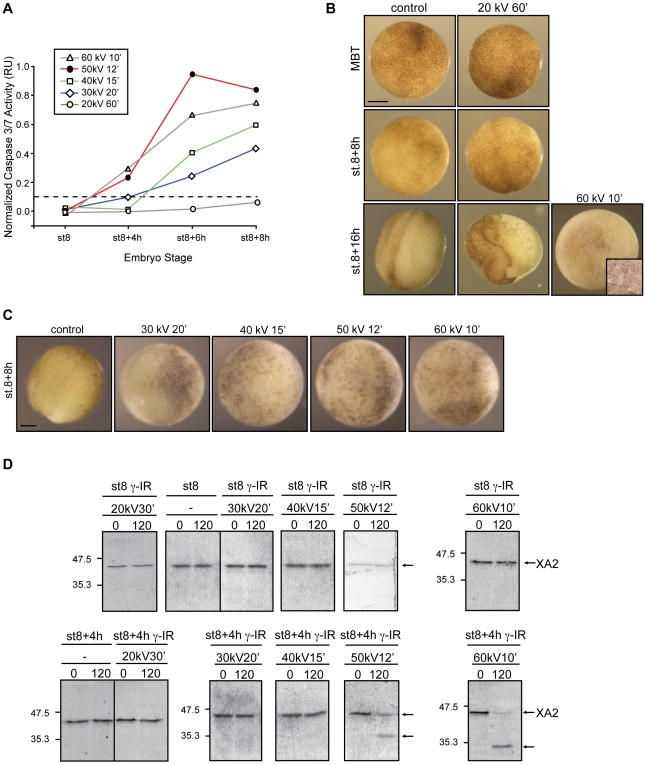
*Xenopus* embryos exhibit different biological responses to the same dose of radiation. **A.** Embryos irradiated (γ-IR) or not (control) before the MBT were collected at the indicated energies and times as summarized in Fig. S2.C. Caspase activity was assayed as described in the legend of Figure 2. Points indicate the average of ten embryos at each time stage. Figure shows data from a single experiment that was repeated three times with similar results. **B.** Morphology of *Xenopus* embryos non-irradiated (control) or irradiated with 20 *kV* for 60 min and collected at MBT (st.8), st.8+8 h and st.8+16 h. For comparison, an embryo treated with 60 *kV* for 10 min and collected at st.8+16 h is displayed. Inset shows a higher magnification image of typical apoptotic morphology from a similar embryo. **C.** Morphology of *Xenopus* embryos non-irradiated (control) or irradiated with 30, 40, 50, 60 *kV* for 20, 15, 12 and 10 min, respectively, and collected 8 h after the MBT. Scale bar, 250 µm. **D.** Caspases 3/7 activity were also assessed by cleavage of the radiolabeled cyclin A2 in extracts from control (non-irradiated) or γ-IR with the same total dose as indicated. Arrows on the right denote radiolabeled *Xenopus* cyclin A2 (XA2) and its cleaved form.

We then look at the effect of radiation on the embryos' fate from a different perspective and ask whether their biological response depends exclusively on the total dose administered to the system. To test this possibility directly, we delivered the *same* total dose to embryos using different photon energies and exposure times (Fig. 5). Specifically, we irradiated embryos with energies equal to 20, 30, 40, 50 and 60 *kV* for 60, 20, 15, 12, and 10 min, respectively. In each case, dose values were determined by microdosimetry using the appropriate calibration of the instrument (Fig. S2.B) as described in the Materials and Methods section. A summary of the experimental dose values for each *kV*xmin combination is presented in Fig. S2.C. In all cases, the *absorbed* dose was of 82.45 Gy±8.4 and therefore comparable (Fig. S2.C).

Samples were collected at the indicated times and analyzed for caspase activity and cyclin A2 cleavage (Fig. 5 and Fig. S1.A). Interestingly, the biological response of the embryo to the same total dose of radiation was dramatically different. For instance, embryos irradiated with 20 *kV* for either 30 or 60 min did not show detectable levels of caspases activation (Fig. 5 and Fig. S1.A), whereas those exposed to 60 *kV* for 10 min exhibited significant activity as early as 4 h after the MBT (Fig. 5.A). Accordingly, the former embryos remained viable and comparable to control non-irradiated samples throughout the time course analyzed, whereas the 60 *kV*-irradiated embryos were apoptotic 8 h after the MBT (Fig. 5.B). Interestingly, we also found “intermediate” states where the embryo eventually developed apoptosis (st.8+8 h) in response to radiation (30, 40, and 50 *kV*) but caspase activation only surpassed a control threshold 6 h after MBT (Fig. 5.A–C).

We then examined the effect of high-dose radiation (∼80 Gy) on late development in embryos treated with low (20 *kV* 30 min and 60 min) and high-energies (60 *kV*, 10 min). Control and radiation-treated embryos were analyzed following external criteria and thus, delimitation of the frontal field by pigment lines and complete closure of the suture of the neural tube were easily recognizable features. Surprisingly, we found that, unlike high-energy-irradiated embryos, embryos treated with low incoming energies were viable even 20 h after the MBT (Fig. 5.B and Fig. S1.B). Interestingly, whereas embryos irradiated with 20 *kV* 30 min exhibited normal development, albeit with a slight degree of delay (Fig. S1.B), others exposed for 60 min showed more dramatic abnormalities roughly 24 h post-fertilization, an event that later developed in early signs of apoptosis (Fig. 5.B). Embryo extracts from viable samples collected 20 h after irradiation with 20 *kV* for 30 min did not exhibit detectable levels of caspase activity as evidenced by the absence of cyclin A2 cleavage (Fig. S1.B). However, same embryos underwent apoptosis or developed various degrees of abnormalities at later times, even when developmental defects prior to gastrulation were not observed. We believe that this is most likely due to accumulation of mutations and the inability of the system to effectively repair the DNA damage (data not shown).

Levels of caspase activity above the threshold irreversibly resulted in embryo death. Samples from blastula, gastrula, and neurula stage embryos treated with various incident energies were analyzed for the cleavage of a specific caspase substrate shortly after irradiation (Fig. 5.D). Cyclin A2 cleavage correlated with caspase activity in 50 and 60 *kV*-irradiated embryos as early as 4 h after the MBT (Fig. 5.D). In agreement with the data presented in Fig. 5.A, cleavage of cyclin A2 was absent in extracts from samples irradiated with energies equal to or lower than 40 *kV* and collected 4 h after the MBT. However, analysis of the samples at later points showed that all but those irradiated with 20 *kV* of energy exhibited cyclin A2 cleavage (data not shown) in agreement with the results shown in Figs. 5.A and B. Thus, the total dose administered to the system *alone* does not determine its response, but, rather the energy of the incoming photon influences the biological outcome of the system.

### Analysis of Cell Death Induced by Augmented Energy Levels

**Figure 6:**
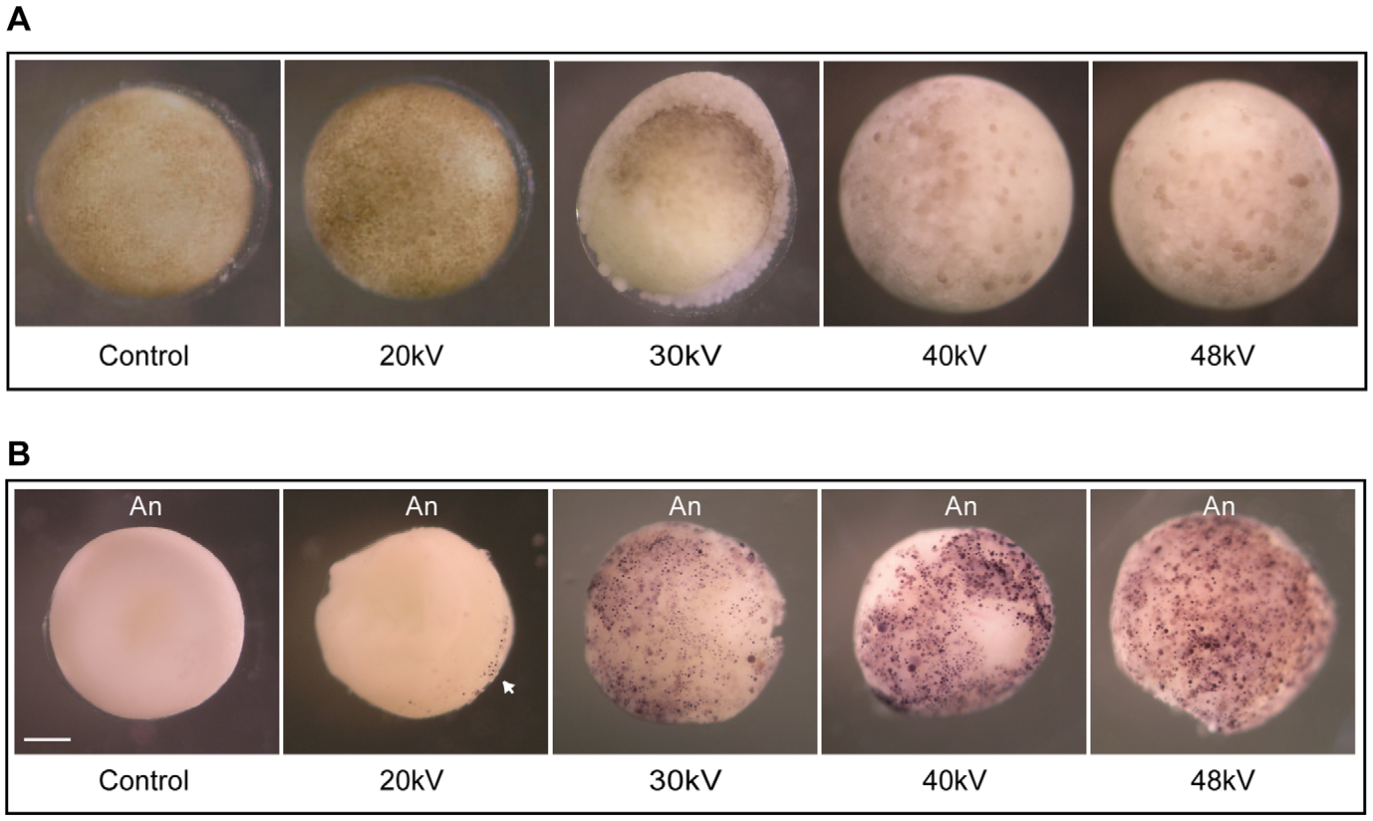
Whole-mount TUNEL assay exposes apoptotic cells in irradiated embryos. **A.** Pre-MBT embryos were exposed to different energies 20 *kV*, 30 *kV*, 40 *kV* and 48 *kV* to equal a total dose of ∼65 Gy. Non-irradiated embryos are referred to as “control”. Six hours after the MBT, embryos were fixed in MEMFA as described in the “Materials and Methods” section and photographed. **B.** TUNEL staining was performed on fixed embryos to detect DNA fragmentation. Embryos were treated as described in (**A**). Intense TUNEL staining was detected in the animal pole portion of the embryos. The embryos shown in **B** are representative of the TUNEL staining observed following analysis of ∼80 embryos of which 20% were stained. Arrowhead points to labeled nuclei. An, animal pole. Scale bar, 250 µm.

We then employed an independent assay to test whether the apoptotic response that results from energy-dependent exposure correlates with DNA damage. With this in mind, we used a highly sensitive indicator of DNA fragmentation *in situ*, whole-mount TUNEL (terminal deoxynucleotidyl transferase-mediated nicked-end labeling) staining, a method that allows the detection of apoptotic cells at high frequency in early embryos (Fig. 6) [33], [34]. Accordingly, embryos were exposed to various energies and times for which the system is above (≥30 *kV*, ∼65 Gy) or below (∼20 *kV*) a threshold response defined in our experimental sample (Figs. 2, 3 and 5). Treated embryos were analyzed for DNA fragmentation 8 h after the MBT (Fig. 6.A). In agreement with previous reports [33], the presence of TUNEL-positive embryos in control samples (non-irradiated) was limited to less than 3% of all embryos analyzed. This event, therefore, represents the normally occurring programmed cell death that is an essential part of embryonic development and that is expected to occur after gastrula stages and during the maturation of the nervous system [33]. Over 95% of embryos treated with energies equal to or greater than 30 *kV* exhibited an extensive pattern of TUNEL-positive cells after the MBT in late blastula that persisted through later stages of development (Fig. 6.B). In no case did we detect the appearance of extensive TUNEL-positive cells in embryos exposed to 20 *kV*; instead, these embryos closely resembled control samples. Two observations are of note here. First, greater incoming energy correlates with the detection of more TUNEL-positive cells on the animal pole of embryos, thus, indicating a larger extent of double strand breaks due to apoptosis (Fig. 6.B). Second, dying cells appear to be randomly distributed in some cases during normal gastrulation and in 20 *kV*-irradiated embryos (Fig. 6.B, arrowhead), an observation that has been previously reported in gastrulating newt, *Cynops pyrrhogaster*, chicken, *Xenopus* and mouse embryos as well [27], [33], [35], [36]. Whereas this event might be part of the normal developmental process in the embryo, the loss of a specific subset of cells as noticed by localized positive TUNEL-staining could well explain some of the phenotypic defects observed in a reduced number of tailbuds and revealed 20 h after exposure of embryos to 20 *kV* (Fig. 6.B, arrowhead).

## Discussion

Our studies are the first direct demonstration that radiation-triggered cell death is susceptible to the energy of the individual photons from an electromagnetic radiation source, such as X-ray, rather than the sole total dose absorbed by the system. Thus, we revisited the concept of dose-dependent induction of apoptosis that is the cornerstone of a full spectrum of current therapies used in malignant diseases (*i.e.*, cancer) and non-malignant conditions (*i.e.*, trigeminal neuralgia, thyroid eye disease, pigmented villonodular synovitis). Importantly, it will most likely affect the precise treatment intent for radiotherapy (*i.e.*, curative, adjuvant, therapeutic, or palliative) depending on the energy of the source, how radiation is administered, and whether it is combined with surgery, chemotherapy, hormone therapy, or a mixture of these.

To begin, we evaluated the contribution of the exposure time and X-ray energies associated with the beam to the cell death process and thus, determine whether the unique factor that influences the fate of the cell is the total amount of energy delivered to the system. We chose a simple *in vivo* system - the *Xenopus laevis* embryo - which provides an effective model to study radiation-mediated apoptosis in early development [26], [30], [31], [32], [37]. Here, the effect of radiation only becomes apparent when embryos are exposed before the MBT and is conspicuous during and after gastrulation when the pluripotent embryonic cells begin to differentiate. First, we tested the effect of increasing the energy of the incoming photon by augmenting the voltage setting while keeping a constant exposure time (Fig. 2). In this scenario, the total dose delivered to the system increases, as does the energy of the photons. Our analysis revels that *i*) embryos exposed to low-energy values (≤20 *kV*) remain viable throughout the time course analyzed, *ii*) the greater the energy of the incident photons and, therefore, the greater the total radiation dose, precipitates earlier apoptotic events in embryos, including morphological hallmarks of apoptosis as well as activation of caspases, and *iii*) exposure time is not a variable in this scenario. We speculate that both the difference of the kinetics of caspase detection and the maximum enzymatic activity observed at the end point of our experiments resulting from increasing the energy of the incident photon are due to decreased attenuation and, therefore, increased penetration since the average distance that photons penetrate a specific material (embryo in this case) is determined by the photon energy, the type of material, and its density. In general, high-energy photons are more penetrating than low-energy photons. This is particularly important if we consider that the *Xenopus* embryo is a multilayer cellular system in early embryogenesis [24] and that penetration will be a critical influence on the number of pluripotent cells damaged at once. In addition, another issue is the amount of energy needed to damage DNA, and how extensive the damage must be for the repair mechanism to signal through apoptosis. This is a question that deserves substantive analysis and that will be revisited later in this section after establishing the contribution of the photon energy and total dose for the embryo response.

**Figure 7:**
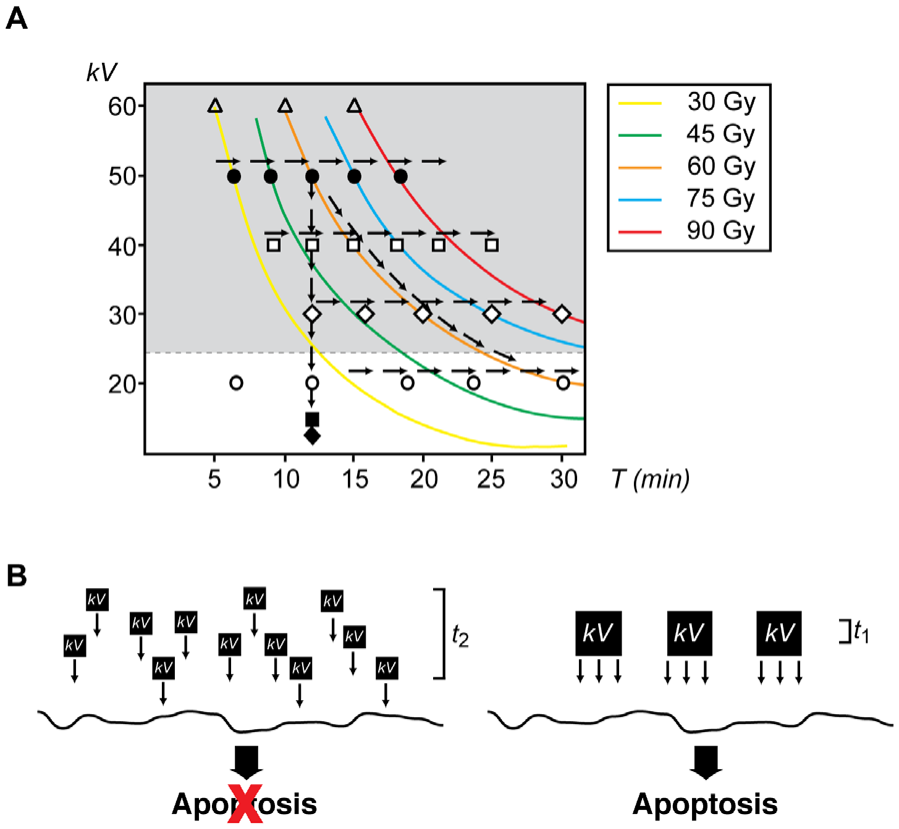
The energy-dependent model of the apoptotic response. **A.** Summary of all experimental conditions analyzed in this work. Arrows indicate each of the three scenarios tested. Energy was measured in kilovolts (*kV*) and exposure time (*T*) in min. Colored lines indicate the same total exposure dose. Symbols indicate various energies tested: ⧫: <10 *kV*, ▪: 10 *kV*, ○: 20 *kV*, ◊: 30 *kV*, □: 40 *kV*, •: 50 *kV*, △: 60 *kV*. **B.** Conceptual model for the contribution of energy and exposure time to the induction of apoptosis. Energy (*kV*) is delivered to the sample in either small (left) or large (right) quantum packages. In our schematic representation, small packages are ¼ the size of the large ones, whereas the exposure time is four times longer (*t_2_* = 4*t_1_*) in the model represented on the left, and thus, the total exposure dose is the same in both settings. In our model, apoptosis is exclusively induced when large packages of energy are delivered to the sample even when the total exposure dose is the same in both scenarios.

Next, we evaluated the consequences of maintaining a constant voltage and, therefore, photon energy, while altering the exposure time of the embryo to radiation (Fig. 3). In this scenario, embryos exposed to 20 *kV* for a period of time ranging from 6 to 60 min remained viable throughout the time course analyzed. Moreover, each experimental condition exemplified a different total dose delivered to the system ranging from 8 Gy, for the lowest exposure time, to ∼80 Gy for samples exposed for up to 60 min. When a similar analysis was performed for embryos exposed to energies above 20 *kV* for various times, the end result was remarkably different, and here the embryos ultimately died of apoptosis (Figs. 3 and 4). The overall data show a trend in which increasing energy values (ε) lead to cell death. As shown in Fig. 2, we favor a model where penetration is an energy-dependent factor that influences the onset of apoptosis. Thus, the level of caspase activity detected early on (st.8+4 h) in high-energy treated samples (50 *kV* and 60 *kV*) depends on the total number of cells undergoing apoptosis at any given time. However, what is truly remarkable in this experiment, as emphasized in Fig. 5, is the fact that for the *same* total dose of radiation, the biological system responds much differently depending on incident energy. For example, when embryos were irradiated with 480 *kV-*min, their response varied from cell progression and differentiation (for 20 *kV*, 24 min) to cell death (for 30 *kV*, 16 min, 40 *kV*, 12 min, ∼50 *kV*, 9 min). This comparison undoubtedly establishes that the energy of the incoming photon, as opposed to the total dose delivered, is the key component of the electromagnetic radiation influencing the fate of the cell. These results also suggest the existence of a threshold value of energy below which cells can be irradiated, even at high doses, and still survive (Fig. 7.A). Further studies are needed to determine accurately the value below which cells tolerate radiation and, more importantly, to uncover the biological implications of low-energy radiation versus low-dose exposure for long-term systems.

DNA double-strand breaks (DSBs) are among the most severe lesions caused by both endogenous and exogenous genotoxic conditions. Naturally occurring DSBs usually arise spontaneously and as a result of several cellular processes including the generation of reactive oxygen species by endogenous metabolites, collapsed replication forks, and during certain specialized processes such as V(D)J recombination (for review see [38]). In other cases, exogenous physical and chemical agents such as ionizing radiation (X- and γ-rays), UV light, topoisomerase inhibitors, and radiomimetic drugs are largely responsible for DSBs and other types of DNA damage [39]. Two major pathways have evolved to repair DNA DSBs in somatic mammalian cells and thereby suppress genomic instability: non-homologous end joining (NHEJ) and homologous recombination (HR) (for reviews see [40], [41]). Depending on the context, both mechanisms may either compete or act together to fix DSBs in eukaryotic cells. Unlike HR where rejoining of the DNA ends requires the presence of a homologous template and is mainly important during the late S and G_2_ phases of the cell cycle, the repair of DSBs by NHEJ requires little or no sequence homology and can occur throughout the cell cycle, although it is of particular importance during G_0_, G_1_, and early S phase [42], [43]. In both cases, the histone 2A family member X (H2AX) is phosphorylated at the C-termini Ser139, a common recognition site of all three major phosphatidyl inositol 3-kinase-like kinases, to form discrete foci at the DSB sites shortly after damage (for review see [38] and references within).

Current knowledge on the effectiveness of radiation to cause DSBs establishes a linear dependency with the radiation dose in which typical yields are between 5–6 DSBs/Gbp/Gy for photon irradiation or what is equivalent to 30–36 DSBs/Gy for a diploid human cell in G1 phase [44]. This is in agreement with recent data that establishes a 1∶1 correlation between γ-H2AX foci and DSBs after irradiation in non-replicating cells [45], [46], [47], [48]. Quantitative values for induction and repair of DSBs at various doses were determined by pulse-field gel electrophoresis (PFGE) and the formation and disappearance of γ-H2AX foci [45], [49], [50], [51]. Remarkably, the induction of γ-H2AX foci/cell for a range of doses between 1 mGy and 100 Gy with a constant energy value is unexpectedly linear as obtained by PFGE measurements [45], [49]. When analyzed in the context of our experiments and from the standpoint of total dose delivered to the system, we could expect that the number of DSBs generated in two given samples (*e.g.* 20 *kV*, 60 min and 60 *kV*, 10 min) will be essentially the same since the total dose in both experimental setups was roughly similar (∼80 Gy for both samples in our example). Surprisingly, we found the biological response of the embryo was remarkably different for the same total dose in different scenarios, as shown in Figure 5, and that this event correlates with both increasing photon energy and augmented DSBs (Fig. 6). Based on these observations, we postulate a parallel to the photoelectric effect in the sense that the outcome of the system depends more sensitively on one parameter, the photon energy, than on the absorbed dose and that the number of DSBs is likely to be different in different scenarios. However, it is of note that the precise number of DSBs occurring in response to radiation depends on additional factors (*e.g.*, surface upon which the cells are grown) that generate secondary electrons with energies similar to the original photon and with quite complex [52], [53].

An additional point of consideration is the efficiency of the repair system to fix DSBs induced in response of various doses of radiation. The kinetic of foci disappearance has been used to gain insights on the efficiency of the DSB repair mechanism in various cellular systems. The direct role of the repair system in radiation-induced DSB has been evaluated at various doses and in confluent cultures of a DSB repair-deficient human primary fibroblast cell line, 180BR [54]. Current PFGE studies demonstrate that *i*) DSB induction and γ-H2AX foci formation are unaltered in 180BR cells when compared with a wild type primary fibroblast cell line (MRC-5); *ii*) 180BR cells exposed with up to 2 Gy foci are lost with slower kinetics than wild type cells; *iii*) there is a decreasing capacity for DSB repair with decreasing dose; *iv*) there is no large differences in induction of DSBs in different genomic locations in response to X-rays; and *v*) the number of DSBs not correctly rejoined after irradiation is essentially the same for high doses f up to 160 Gy [45], [49], [50], [54]. In addition, PFGE studies have shown that the time course for DSB repair in primary human fibroblasts is independent of the initial dose of X-rays for values grater than 10 Gy [49]. However, the same cellular system seems to follow a much slower kinetics of DSB repair when cells are exposed to very low doses of X-ray radiation and thus the distribution of cells with a given number of foci does not change for repair times up to 24 h [45]. Thus, it seems unlikely that the capacity of the DNA repair mechanism would be responsible for the differences observed in response to high-energy irradiation in our system. The details of the events that follow the exposure of the embryo to an initial photon of a particular energy (ε) and from there to the generation of secondary electrons, DSB, and apoptosis is a highly non-trivial undertaking that is beyond the scope of this work. Instead, we explore the possibility of a parallel in biology to the photoelectric effect; namely, that one of the three components (photon energy ε, photon current *j*, exposure time *T*) associated with the exposure dose plays a determinant role in inducing apoptosis. Here, we propose that in embryos, the extent of DNA-damage increases when exposed to radiation with higher photon energies even if the total dose absorbed is the same (Fig. 7.B).

An interesting observation refers to results that show that DSBs induced by photon energies ranging from 15 to 660 *kV* are repaired with similar kinetics and have similar dependencies upon checkpoint components in primary human fibroblasts [55]. Even though at a first glance, these data seem to conflict with our model, it does not consider the remodeling nature of the cell cycle in the early *Xenopus* embryo upon reaching the MBT. Cell division in pre-MBT embryos alternates rapid rounds of DNA synthesis and mitosis (∼20–30 min) with no discernible G phases [56], [57]. Thus, pre-MBT embryos lack checkpoints that halt the cell cycle in response to DNA damage [30], [32] and are unable to trigger cell death. Gap phases are established after the MBT when the cell cycle lengthens, somatic-like checkpoints are functional, and cells arrest in response to damage [30], [31], [56], [57], [58]. In our experiments, embryos were irradiated before the MBT; thus, the energy-dependent increase of DNA fragmentation (Fig. 6) observed for a given dose of radiation reflects the inability of the system to repair the damage before cell cycle transition occurs. Therefore, the *Xenopus* embryo represents the most suitable system to directly assess the contribution of each component of electromagnetic radiation.

## Supporting Information

Figure S1 A. Embryos were irradiated (γ-IR) or not (control) before the MBT (st.6) with either 20 kV of energy for either 30 or 60 min, collected at st.8 (MBT) and 4, 6 and 8 h after the MBT, and frozen. Samples equivalent to ten embryos were tested for caspases 3/7 activity using a specific colorimetric substrate as described in the “Materials and Methods” section and normalized as described in the legend of Fig. 2. Points indicate the average of ten embryos at each time stage. Results similar to those presented here were observed in two independent experiments. B. Morphology of Xenopus embryos not irradiated (control) or irradiated with 20 kV for 30 min and collected at 8 h and 20 h after the MBT. Scale bar, 250 µm. Xenopus cyclin A2 cleavage assay is shown on the right. Arrow indicates cleavage product.
(1.88 MB TIF)

Figure S2 Dosimetry measurements. A. Each TLD card contains four pellets. Three measurements were performed for each radiation treatment as indicated (T1–3) in the left panel. In some experiments cards were placed on top (right card labeled “top”) or underneath the embryos (middle card labeled “bottom”) and exposed to various beam energies (20, 30, 40, 50, 60 kV) for the indicated experimental times. B. Each experimental measurement (T1–T3) is converted to Gy's and averaged based on the instrument's calibration (30 kV for 10 min corresponds to a dose of 37 Gy). Average values and standard deviations for “top” cards, are shown for a range of energies (kV) and times (min). To emphasize the rationale behind our choice of these parameters, we have an additional column (kV-min) showing each energy and time combination correspond to the same total amount of energy delivered by the beam. Note that all absorbed doses are essentially the same with the exception of the 20 kV case which shows approximately half the dose when compare with the others. Ratios of these doses, relative to the calibrated case, are shown in the 5th column. C. Range of energies (kV's) and times (min) used for the experiments shown in Fig. 5.A.
(1.60 MB TIF)
