# Supplementary figures and images for: Shorter Exposures to Harder X-Rays Trigger Early Apoptotic Events in Xenopus laevis Embryos
Source: PLoS One. 2010 Jan 29;5(1):e8970. doi: 10.1371/journal.pone.0008970 (PMC2813296; doi:10.1371/journal.pone.0008970)

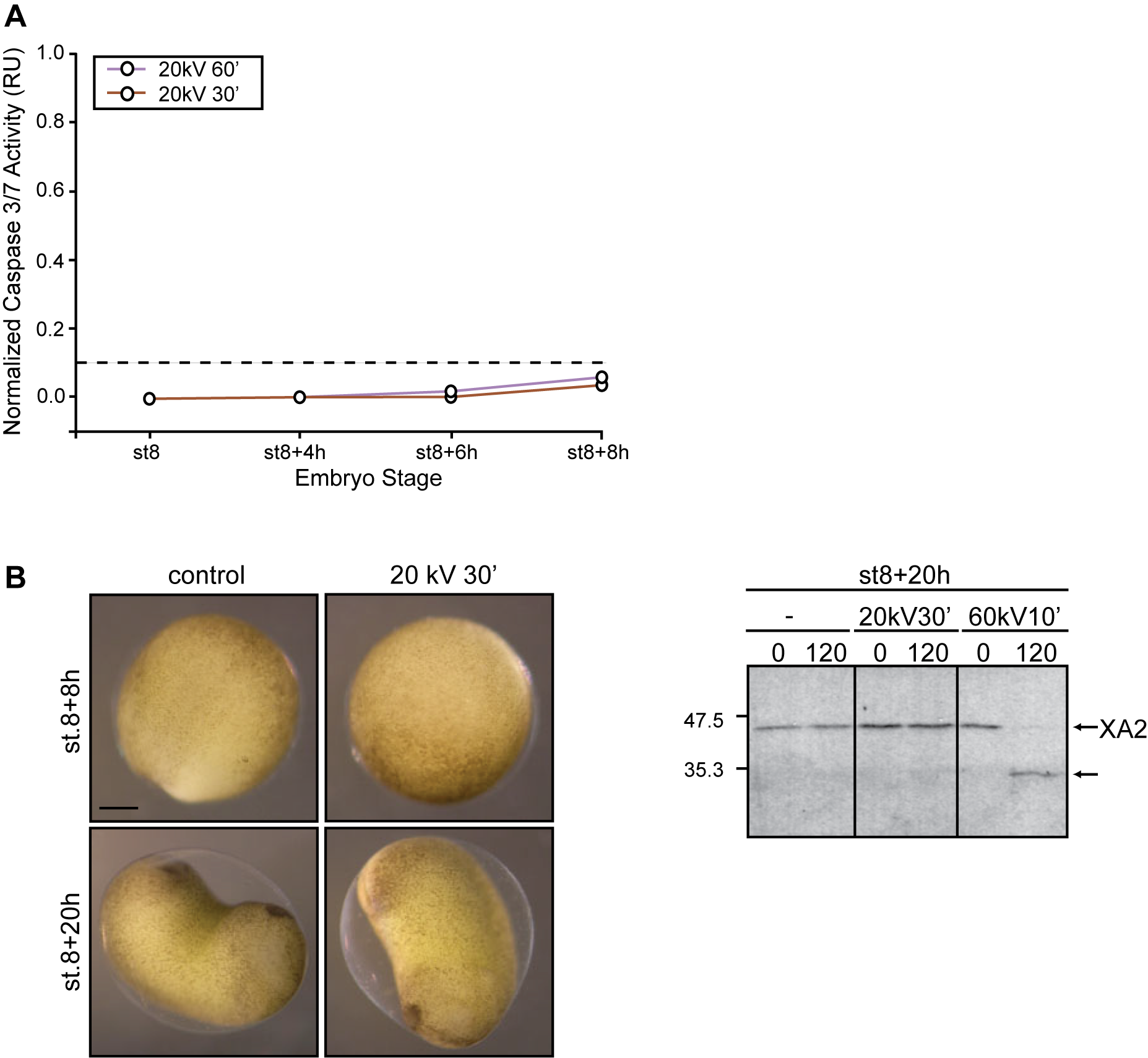

Supplement: Figure S1 — A. Embryos were irradiated (γ-IR) or not (control) before the MBT (st.6) with either 20 kV of energy for either 30 or 60 min, collected at st.8 (MBT) and 4, 6 and 8 h after the MBT, and frozen. Samples equivalent to ten embryos were tested for caspases 3/7 activity using a specific colorimetric substrate as described in the “Materials and Methods” section and normalized as described in the legend of Fig. 2. Points indicate the average of ten embryos at each time stage. Results similar to those presented here were observed in two independent experiments. B. Morphology of Xenopus embryos not irradiated (control) or irradiated with 20 kV for 30 min and collected at 8 h and 20 h after the MBT. Scale bar, 250 µm. Xenopus cyclin A2 cleavage assay is shown on the right. Arrow indicates cleavage product. (1.88 MB TIF) [file pone.0008970.s001.tif]

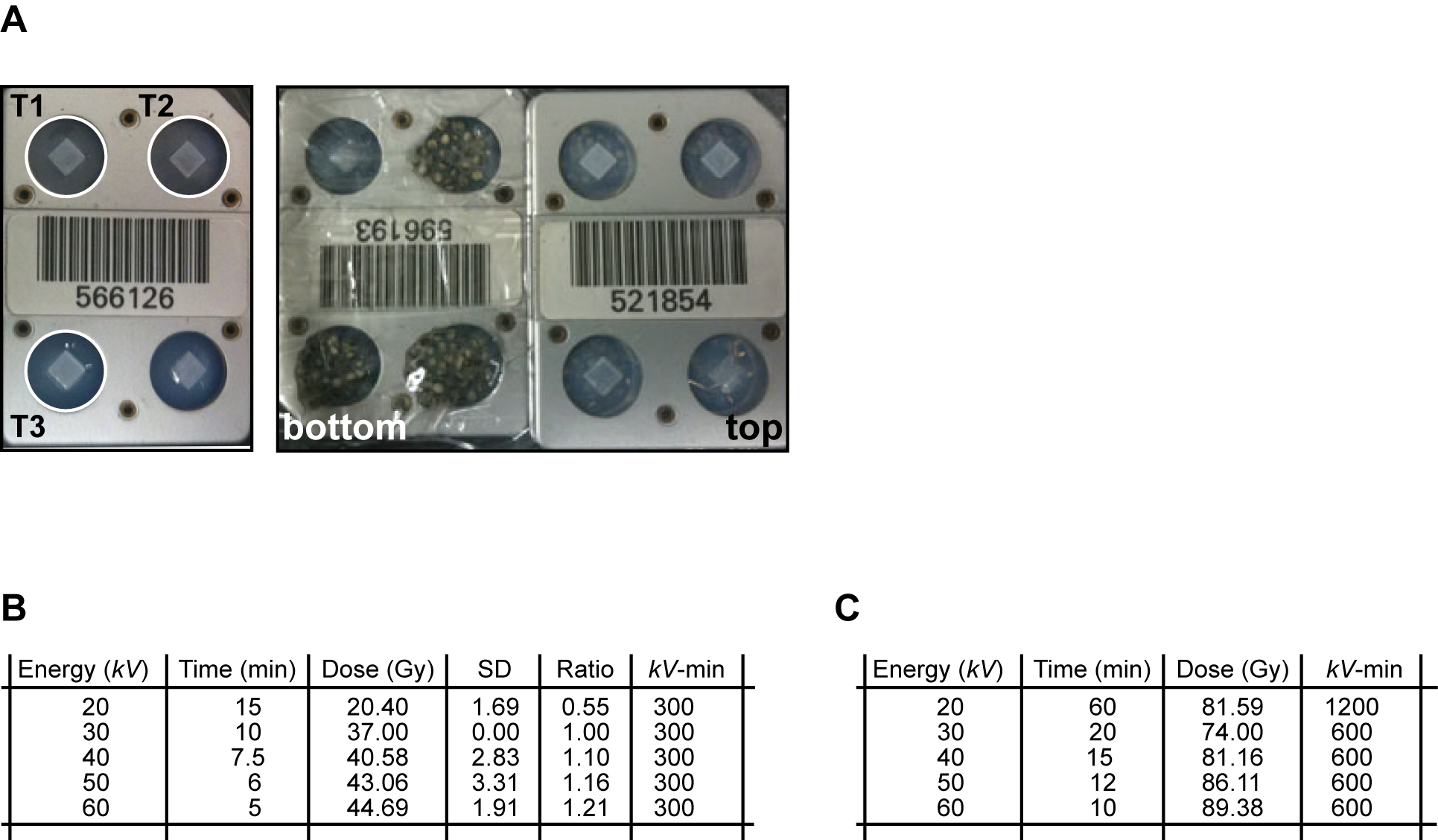

Supplement: Figure S2 — Dosimetry measurements. A. Each TLD card contains four pellets. Three measurements were performed for each radiation treatment as indicated (T1–3) in the left panel. In some experiments cards were placed on top (right card labeled “top”) or underneath the embryos (middle card labeled “bottom”) and exposed to various beam energies (20, 30, 40, 50, 60 kV) for the indicated experimental times. B. Each experimental measurement (T1–T3) is converted to Gy's and averaged based on the instrument's calibration (30 kV for 10 min corresponds to a dose of 37 Gy). Average values and standard deviations for “top” cards, are shown for a range of energies (kV) and times (min). To emphasize the rationale behind our choice of these parameters, we have an additional column (kV-min) showing each energy and time combination correspond to the same total amount of energy delivered by the beam. Note that all absorbed doses are essentially the same with the exception of the 20 kV case which shows approximately half the dose when compare with the others. Ratios of these doses, relative to the calibrated case, are shown in the 5th column. C. Range of energies (kV's) and times (min) used for the experiments shown in Fig. 5.A. (1.60 MB TIF) [file pone.0008970.s002.tif]
